# From Health Checkups to Mental Health Referrals: Developing an AI-Based Depression Screening Model Using Routine Data in Older Adults Living Alone

**DOI:** 10.1192/j.eurpsy.2026.11847

**Published:** 2026-07-31

**Authors:** H. W. Jung, J. J. Lee, O. Kim

**Affiliations:** 1 Department of Psychiatry; 2 Digital Mental Health Innovation Center, Dankook University, Cheonan-si, Korea, Republic Of

## Abstract

**Introduction:**

Depression in older adults often remains underdiagnosed due to somatic symptoms, stigma, and low self-recognition. This challenge is particularly pronounced among those living alone, who face social isolation and limited access to mental-health care. To address this gap, an AI-based screening model using routine health checkup data—termed the *Depression Prediction based on Anthropometric and Clinical Signs (DEPACS)* framework—was developed to support early detection and referral in community settings (Figure 1).

**Objectives:**

This study aimed to develop and validate an explainable AI model capable of predicting depression risk among older adults living alone using non-psychological, routinely collected health data, and to compare its predictive performance with a PHQ-9–based model.

**Methods:**

Data were obtained from 1,087 participants in the *Intensive Managed Care for Elderly Living Alone (IMCELA)* program in Chungcheongnam-do, South Korea. Two models were trained:

1. a PHQ-9–based model to validate predictive performance, and
2. a DEPACS-based screening model using seven domains (socioeconomic, vital, anthropometric, physical, behavioral, chronic disease, and sleep quality).

Five algorithms—SVM, Random Forest, Gradient Boosting, XGBoost, and KNN—were optimized via grid search and 5-fold cross-validation. AUROC and AUPRC evaluated performance, and SHapley Additive exPlanations (SHAP) visualized feature importance.

**Results:**

The PHQ-9–based model achieved excellent discrimination (AUROC > 0.98) across algorithms (Figure 2). SHAP analysis revealed that sleep disturbance, fatigue, anhedonia, and poor concentration were the strongest predictors, followed by subjective memory complaints and chronic pain.

In contrast, the DEPACS model showed fair accuracy (AUROC = 0.74; AUPRC = 0.73). Key predictors included sleep quality, waist circumference, balance, age, and exercise (Figure 3). Poor sleep quality and elevated heart rate increased depression risk, whereas greater balance ability and higher waist circumference reduced it. These findings indicate that routine physiological and lifestyle indicators can capture meaningful depressive patterns without explicit psychological items.

**Image 1:**
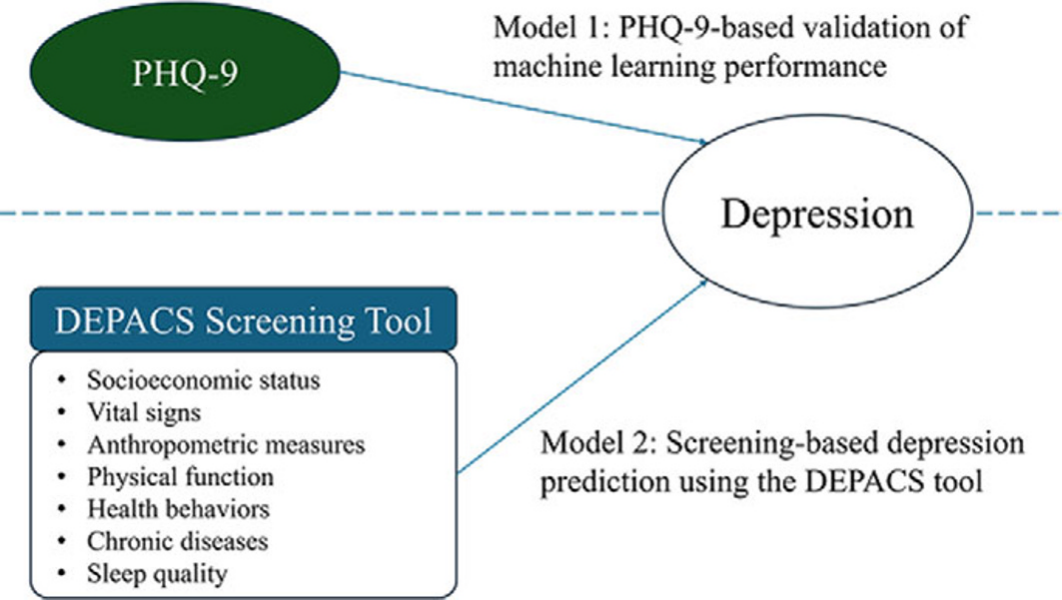

**Image 2:**
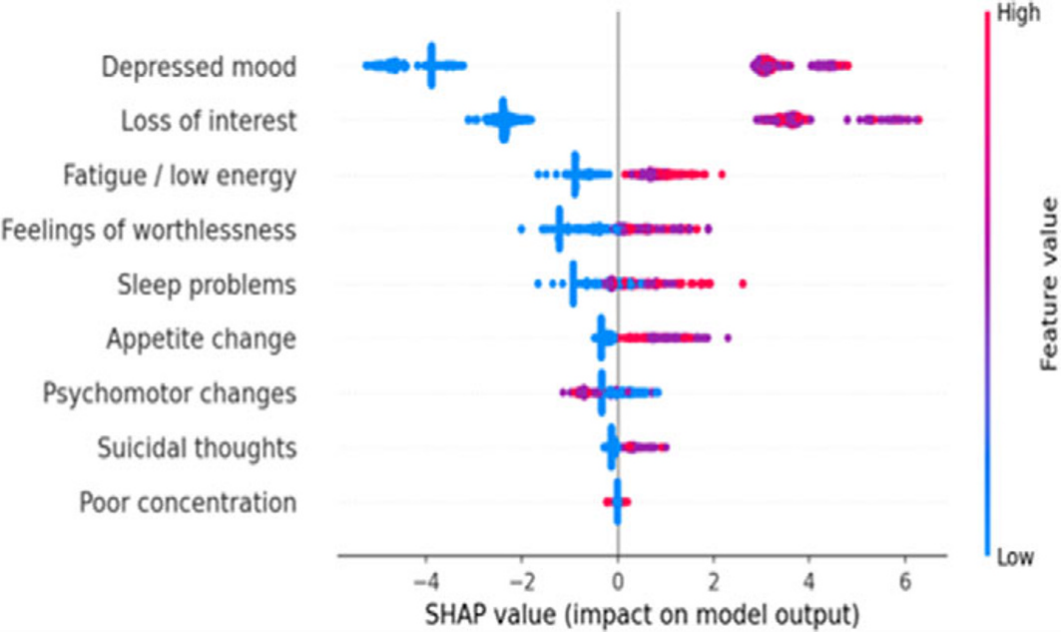
Image 2: Long description.

**Image 3:**
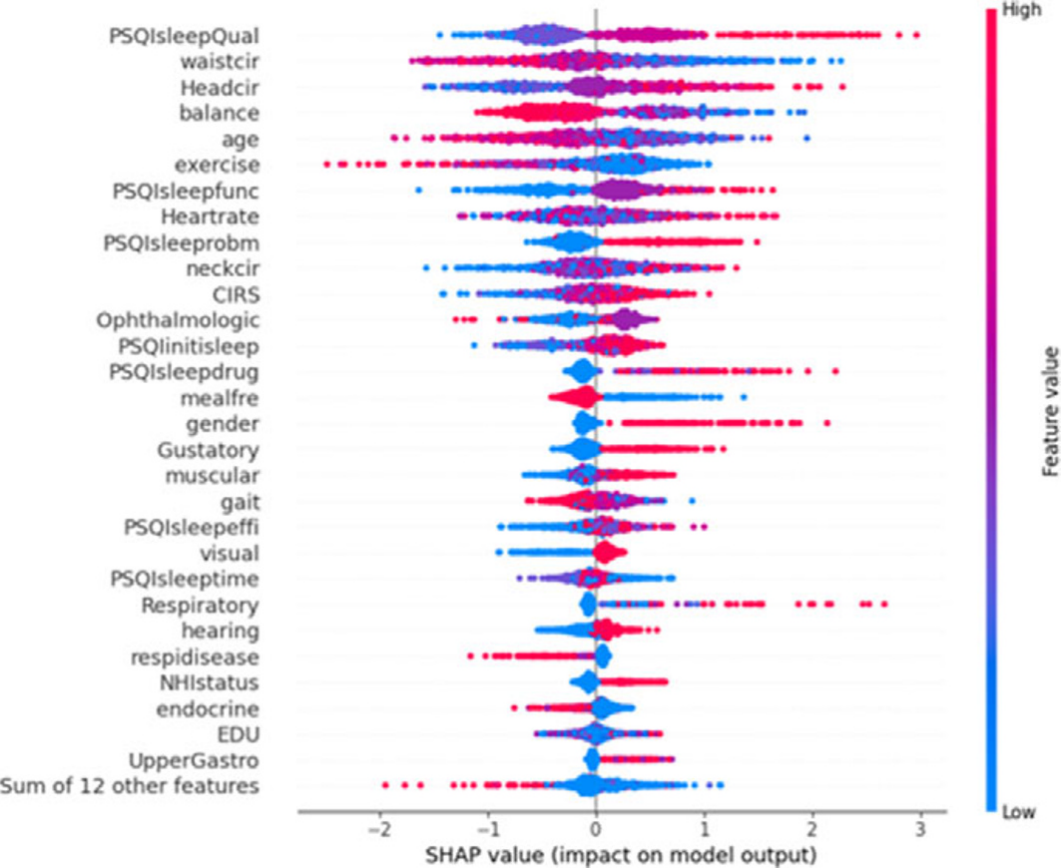
Image 3: Long description.

**Conclusions:**

The DEPACS framework demonstrated that depression risk among older adults living alone can be predicted using only routine health data. Although its performance was lower than the PHQ-9–based model, its interpretability and scalability make it suitable for integration into national health screening programs. This approach offers a cost-efficient, explainable AI solution bridging physical and mental-health assessment in community-dwelling elderly populations.

**Disclosure of Interest:**

None Declared

## Long descriptions

### Image 2: Long description

A dot plot with a vertical zero line. The x-axis is labeled S H A P value (impact on model output) ranging from negative 4 to 6. The y-axis lists nine symptoms from top to bottom. A color scale on the right indicates Feature value from Low (blue) to High (red).

* Depressed mood: Blue dots are clustered around negative 4 and negative 5. Red dots are clustered between 3 and 5.

* Loss of interest: Blue dots cluster around negative 2.5. Red dots spread from 3 to 6.

* Fatigue / low energy: Blue dots cluster around negative 1. Red dots spread from 0 to 2.

* Feelings of worthlessness: Blue dots cluster around negative 1. Red dots spread from 0 to 2.5.

* Sleep problems: Blue dots cluster around negative 0.5. Red dots spread from 0 to 3.

* Appetite change: Blue dots cluster at 0. Red dots spread from 0 to 2.5.

* Psychomotor changes: Blue and red dots are mixed, mostly clustered around 0 with a small spread to negative 1 and positive 1.

* Suicidal thoughts: Blue dots cluster at 0. Red dots spread from 0 to 1.

* Poor concentration: Blue and red dots are tightly clustered at 0.

Navigate back to Image 2:.

### Image 3: Long description

A S H A P summary plot with the x-axis labeled S H A P value impact on model output ranging from negative 2 to 3. The y-axis lists 28 features from top to bottom.

* P S Q I sleep Qual.

* waistcir.

* Headcir.

* balance.

* age.

* exercise.

* P S Q I sleepfunc.

* Heartrate.

* P S Q I sleepprobm.

* neckcir.

* C I R S.

* Ophthalmologic.

* P S Q I initisleep.

* P S Q I sleepdrug.

* mealfre.

* gender.

* Gustatory.

* muscular.

* gait.

* P S Q I sleepeffi.

* visual.

* P S Q I sleeptime.

* Respiratory.

* hearing.

* respidisease.

* N H I status.

* endocrine.

* E D U.

* Upper Gastro.

* Sum of 12 other features.

Each feature has a horizontal distribution of dots. A vertical color bar on the right indicates feature value, where pink represents High and blue represents Low. For P S Q I sleep Qual, high pink values correlate with positive S H A P values, while for exercise, high pink values correlate with negative S H A P values.

Navigate back to Image 3:.

